# Extracellular Vesicle Separation Techniques Impact Results from Human Blood Samples: Considerations for Diagnostic Applications

**DOI:** 10.3390/ijms22179211

**Published:** 2021-08-26

**Authors:** Theophilos Tzaridis, Daniel Bachurski, Shu Liu, Kristin Surmann, Felix Babatz, Manuela Gesell Salazar, Uwe Völker, Michael Hallek, Ulrich Herrlinger, Ina Vorberg, Christoph Coch, Katrin S. Reiners, Gunther Hartmann

**Affiliations:** 1 Institute of Clinical Chemistry and Clinical Pharmacology, University Hospital Bonn, 53127 Bonn, Germany; ttzaridis@sbpdiscovery.org (T.T.); ccoch@uni-bonn.de (C.C.); Gunther.Hartmann@uni-bonn.de (G.H.); 2 Division of Clinical Neurooncology, Department of Neurology, University Hospital Bonn, 53127 Bonn, Germany; Ulrich.Herrlinger@ukbonn.de; 3 Tumor Initiation & Maintenance Program, Sanford Burnham Prebys Medical Discovery Institute, La Jolla, CA 10901, USA; 4 Center for Integrated Oncology Cologne-Bonn, Department I of Internal Medicine, University Hospital of Cologne, 50937 Cologne, Germany; daniel.bachurski@uk-koeln.de (D.B.); Michael.hallek@uk-koeln.de (M.H.); 5 CECAD Center of Excellence on “Cellular Stress Responses in Aging-Associated Diseases”, Center for Molecular Medicine Cologne, University of Cologne, 50931 Cologne, Germany; fbabatz@uni-koeln.de; 6 German Center for Neurodegenerative Diseases (DZNE e.V.), 53127 Bonn, Germany; shu.liu@bfr.bund.de (S.L.); Ina.Vorberg@dzne.de (I.V.); 7 Department of Functional Genomics, Interfaculty Institute for Genetics and Functional Genomics, University Medicine Greifswald, 17475 Greifswald, Germany; kristin.surmann@uni-greifswald.de (K.S.); gesell@uni-greifswald.de (M.G.S.); uwe.voelker@uni-greifswald.de (U.V.)

**Keywords:** extracellular vesicles diagnostics, serum biomarker, plasma biomarker, extracellular vesicle isolation, methods in liquid biopsy

## Abstract

Extracellular vesicles (EVs) are reminiscent of their cell of origin and thus represent a valuable source of biomarkers. However, for EVs to be used as biomarkers in clinical practice, simple, comparable, and reproducible analytical methods must be applied. Although progress is being made in EV separation methods for human biofluids, the implementation of EV assays for clinical diagnosis and common guidelines are still lacking. We conducted a comprehensive analysis of established EV separation techniques from human serum and plasma, including ultracentrifugation and size exclusion chromatography (SEC), followed by concentration using (a) ultracentrifugation, (b) ultrafiltration, or (c) precipitation, and immunoaffinity isolation. We analyzed the size, number, protein, and miRNA content of the obtained EVs and assessed the functional delivery of EV cargo. Our results demonstrate that all methods led to an adequate yield of small EVs. While no significant difference in miRNA content was observed for the different separation methods, ultracentrifugation was best for subsequent flow cytometry analysis. Immunoaffinity isolation is not suitable for subsequent protein analyses. SEC + ultracentrifugation showed the best functional delivery of EV cargo. In summary, combining SEC with ultracentrifugation gives the highest yield of pure and functional EVs and allows reliable analysis of both protein and miRNA contents. We propose this combination as the preferred EV isolation method for biomarker studies from human serum or plasma.

## 1. Introduction

Extracellular vesicles (EVs) are small, membrane-coated particles released by cells with heterogeneous morphological and functional properties. Based on their different release mechanisms and size, EVs can be categorized into small vesicles (small EVs) that arise from a fusion of multivesicular endosomes with the plasma membrane (sometimes termed “exosomes”) and larger vesicles that are released through direct budding of the plasma membrane or via yet unknown pathways involving multivesicular bodies (sometimes termed “microvesicles”) [1]. Despite attempts to set rigorous cut-off values for the size of these two types of EVs, some overlap exists. Microvesicles (or large EVs) range from 100 to 1000 nm and small EVs from 30 to 150 nm [2,3,4]. EVs are known to significantly contribute to intercellular communication via the functional transfer of nucleic acids (DNA, mRNA, miRNA) and proteins [2]. Especially in oncology, EVs play a major role in the interaction between the tumor and the microenvironment and are involved in the regulation of cell proliferation, migration, immunosuppression, and angiogenesis [5,6,7,8,9].

Since EVs can be secreted into the bloodstream and their molecular content is protected from enzymatic degradation, they have been used as a tool for collecting liquid biopsies of various tumor entities. EV-liquid biopsy strategies have included the detection of nucleic acids (mainly RNA but also DNA) and proteins [10,11,12]. Through the continuous improvement of purification methods, there are now numerous techniques available for EV separation and concentration from both serum and plasma, with the most prominent ones being differential ultracentrifugation (UC) and size-exclusion chromatography (SEC) [13]. Differential ultracentrifugation has long been considered the standard method for EV separation and leads to an intermediate, but also unspecific, recovery rate [13,14]. Unfortunately, this technique is time-consuming and shows low reproducibility depending on the protocol and equipment used, thus rendering it impractical for routine diagnostic laboratories [15,16]. In addition, it has been reported that UC can impact the integrity of the isolated EVs and can lead to aggregation [17], which might affect downstream analyses [18]. SEC, especially in combination with a second method (such as filtration or density gradient), leads to higher EV purity but sometimes also a lower recovery of EVs [13]. In contrast to UC, SEC is a fast and highly reproducible method, which does not require special equipment and does not damage EVs [19]. Due to the high dilution factor of this method, various techniques for concentrating EV have been proposed (ultrafiltration (UF), UC, or co-precipitation) [20,21,22], but so far, there has been no clear definition of the most suitable method of EV isolation for specific downstream applications (e.g., RNA analysis or protein detection).

A relatively novel method of EV separation is immunoprecipitation (IP), which captures proteins on the surface of the EVs, either nonspecifically using standard EV markers (CD9, CD63, and CD81) [23] or specifically based on antigens of a tumor entity (e.g., EGFR or CD44 on glioblastoma EV) [24,25]. In both cases, the high affinity between the antibody and antigen makes it difficult to dissociate the EVs from the antibody [22], which leads to limitations in subsequent functional analyses. In addition, EV quantification and quality assessment by nanoparticle tracking analysis (NTA), protein quantification, and flow cytometry are compromised.

In the past decade, a wide array of methods for EV isolation from serum has been developed and commercialized. An extensive number of publications comparing different EV separation techniques have shown that the results of downstream EV analyses vary depending on the method used [13,14,26]. Most of these studies focus on the characterization of a respective molecular biopolymer, such as miRNA or protein. So far, there has been no comprehensive evaluation of the most time-efficient and easily standardized EV-purification strategies for both plasma and serum that considers both the nucleic acid and protein contents. However, it is very likely that in a clinical setting, a combination of information on EV-derived nucleic acids and proteins is necessary, to gain reliable prognostic information.

Here, we have conducted an extensive comparison of different methods to separate small EVs from serum and plasma, including (1) UC; (2) SEC followed by (a) UC, (b) UF, or (c) precipitation (ExSp); and (3) IP. We characterized the vesicles resulting from the different isolation and enrichment strategies by flow cytometry, RNA analysis, nanoparticle tracking analysis (NTA), and immunodetection. The ability of the isolated EVs to deliver functional cargo to target cells was tested using a reporter system of EV-associated induction of prion-like protein aggregates in mouse neuroblastoma cells.

## 2. Results

**Figure 1:**
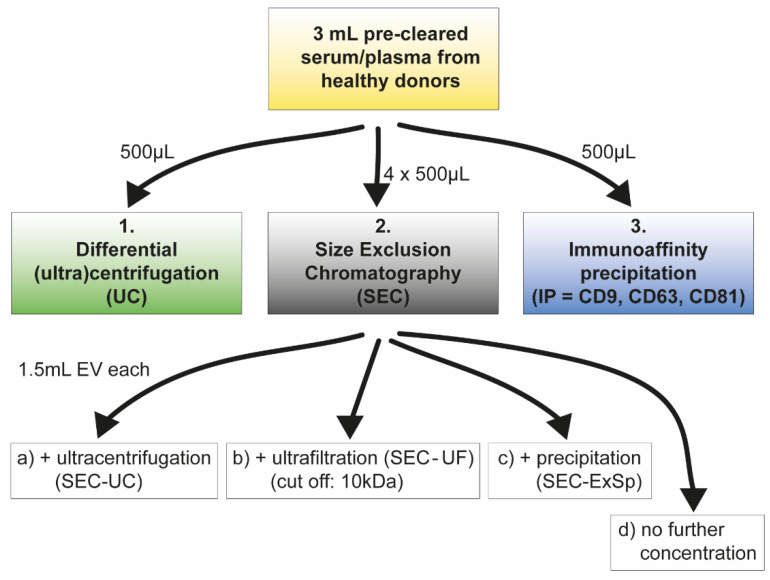
Schematic summary of EV isolation workflow. A total of 3 mL serum or plasma was precleared and split into 500 µL aliquots for subsequent EV isolation with (1) differential (ultra)centrifugation (UC), (2) size exclusion chromatography (SEC), or (3) immunoaffinity precipitation (IP). SEC was performed four times with 500 µL plasma/serum each. Collected fractions (8–10) were first pooled (4 × 1.5 mL = 6 mL), and 1.5 mL aliquots EV suspension was used for each following concentration step.

To identify the most suitable EV isolation method for plasma and serum samples with respect to sensitivity, reproducibility, and feasibility, we conducted a comprehensive comparison of different methods for EV separation and enrichment. The strategies applied were: (1) UC; (2) SEC followed by (a) UC, (b) UF, or (c) ExSp; and (3) IP (Figure 1). To evaluate the different EV isolation strategies, we performed an extensive analysis of the separated vesicles, including nanoparticle tracking analysis (NTA), immunodetection, flow cytometry, RNA analysis, and functional assays.

### 2.1. Particle and Protein Concentration of Serum EVs Separated by UC or SEC

**Figure 2:**
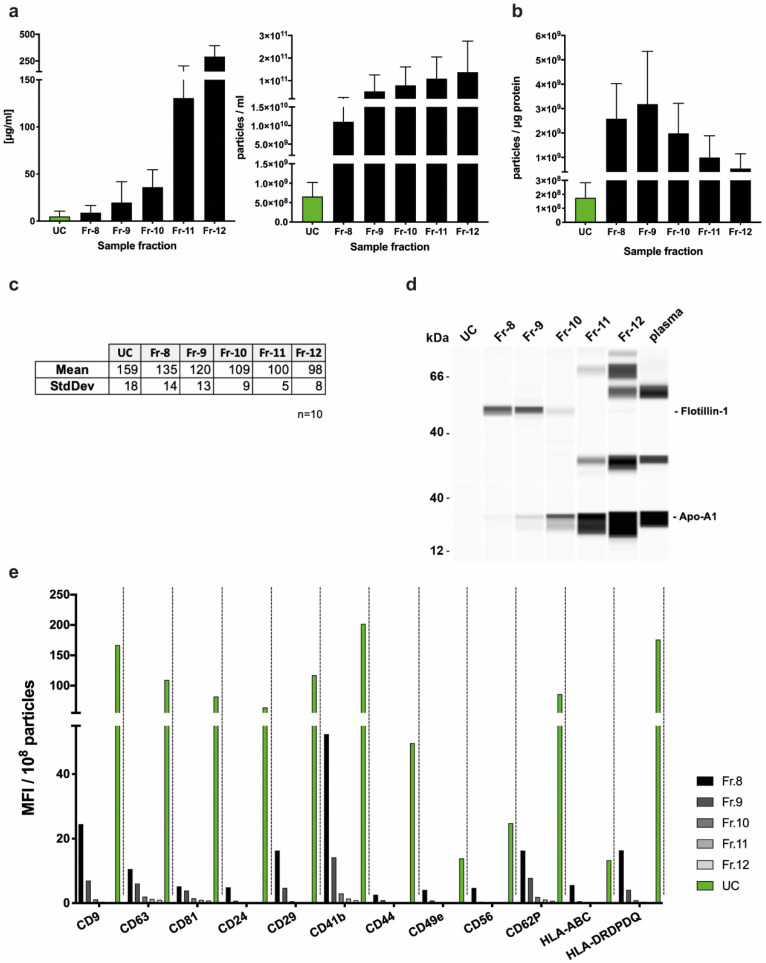
EV yield and characteristics of plasma-derived EV isolated by UC or SEC. (**a**) Yield of EVs from UC-EVs and SEC-EV fractions 8–12 was determined by overall protein concentration (micro-BCA assay) and particle concentration (determined by NTA). (**b**) Calculation of the particle number per µg protein, which shows SEC fractions 8–10 to be the EV-containing fractions. (**c**) Nanoparticle tracking analysis (NTA) of isolated EVs demonstrated that EV size was within the expected range of 100–160 nm. (**d**) Immunodetection of the EV marker flotillin-1 (and TSG101, Figure S1a) and Apo-A1 as indicator for lipoprotein co-isolation. (**e**) Surface protein analysis with the MACSPlex assay. Depicted is one representative experiment of 10. The signals are normalized to particle numbers. (MFI—mean fluorescent intensity, SEC—size exclusion chromatography, UC—ultracentrifugation).

EVs from the plasma of healthy donors were extracted using UC and SEC. For the qEV-column-based SEC samples, fractions 8–12 were collected and analyzed for particle number, size, and protein content. Both particle number and protein concentration were higher in all five fractions collected with SEC compared to UC (Figure 2a).

While the SEC particle number gradually increased with the fraction in a linear manner, the protein concentration was greatly increased in fractions 11 and 12 compared to the earlier fractions and UC. The highest ratio of EV particles to protein units, suggested as a surrogate marker for EV purity [26], was observed in fractions 8–10 (Figure 2b). Size determination with nanoparticle tracking analysis (NTA) showed that the EVs obtained by isolation via UC comprise a larger and more heterogeneous population (159 ± 18 nm), while the size of SEC-EV decreases gradually with the fraction number (135–98 nm) (Figure 2c).

Simple Western immunodetection confirmed the presence of the EV markers flotillin-1 (Figure 2d) and TSG101 (Figure S1a) in SEC-EV fractions 8–10, confirming that these fractions contain EV. Almost no signal was obtained for fractions 11 and 12, suggesting no relevant numbers of EVs to be present in these fractions. The unspecific bands in fractions 11 and 12 emphasize the high levels of non-vesicular proteins, which corresponds to the increasing amount of apolipoprotein A1 (Apo-A1) (Figure 2d). While Apo-A1 was not detectable in the UC-EV sample, the protein was found in the SEC fractions in increasing amounts. Nonetheless, Apo-A1 was reduced up to 130,000-fold (fraction 8) compared to plasma concentrations (Figure S1a). There was no significant protein signal from calnexin (a marker of vesicles originating from the endoplasmic reticulum) in any of the fractions. UC-EVs only gave a weak signal for TSG101 and no signal for flotillin-1, most likely because they were below the limit of detection owing to the low concentration of EVs (Figure S1a).

We assessed the expression of specific proteins on the surface of EVs that were from the different SEC fractions by MACSPlex flow cytometry (e.g., CD9, CD63, CD81) and compared their profile to that of UC-EVs. Fraction 9 of the SEC eluate exhibited the highest overall mean fluorescence intensity (MFI), an indicator of the presence of these proteins (Figure S1b). However, when this was normalized to the number of particles present, we found that UC-EVs have a stronger signal/particle ratio than SEC-purified EVs and that the signals for fraction 8, in all cases, exceed those in fraction 9 (Figure 2e and Figure S1c).

Based on these results, we chose to pool the EV-containing fractions 8–10 for further analyses, to obtain the highest yield and to cover the complete range of EV populations.

### 2.2. All Methods Are Capable of Isolating Small Pure EVs

Since the EVs essentially get diluted three-fold upon elution from the SEC column, the resulting fractions contain EVs with far too low a concentration to be measured in certain assays. Therefore, concentrating the SEC-EVs might be beneficial to prevent potentially significant but very low abundance proteins or nucleic acids being overlooked. Therefore, we added a concentration step after SEC-based EV isolation. We compared three different concentration strategies: ultracentrifugation (SEC-UC), ultrafiltration (SEC-UF), and precipitation utilizing Exo-spin^TM^ buffer (SEC-ExSp).

**Figure 3:**
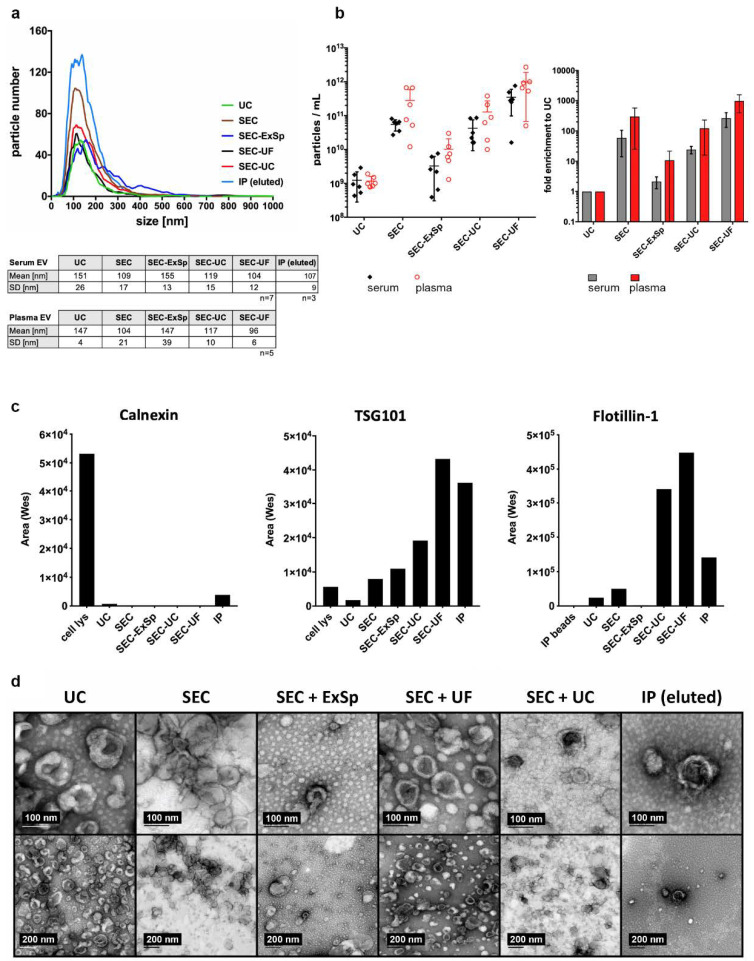
Characterization of EVs isolated and enriched with different strategies. (**a**) NTA of EVs isolated by UC, SEC, SEC-ExSp, SEC-UC, SEC-UF, or IP demonstrated comparable particle sizes for all EVs isolated from plasma or serum. Depicted is one representative experiment. (**b**) Quantitative analysis of EVs by NTA showed the lowest concentration for UC-EVs and greatest loss of EVs (after SEC) for the SEC-ExSp combination (**left**); fold enrichment of particle concentration compared to UC. (**right**) (**c**) Simple Western immunodetection of TSG101, flotillin-1, and calnexin demonstrated strong EV marker enrichment in SEC-UC-EVs and SEC-UF-EVs and confirmed the purity of the EVs based on the lack of the ER protein calnexin. Figure S2 shows the corresponding Simple Western immunodetection image. (**d**) Transmission electron microscopy shows a typical shape and size for EVs in all samples, irrespective of the isolation or enrichment strategy used.

UC alone, SEC with and without subsequent concentration, and immunoprecipitation resulted in the isolation of EVs with a comparable size distribution (Figure 3a). In contrast, the yield and recovery of EVs were strongly method-dependent, UC having the lowest recovery efficacy. Total EV count revealed a loss of SEC-EVs with each of the concentration methods used. SEC-UF gave the highest recovery efficacy of SEC-EVs, while SEC-UC and SEC-ExSp resulted in a substantial loss of EVs (Figure 3b, left graph). Still, the EV concentration was considerably increased after ultrafiltration and ultracentrifugation (mean enrichment: SEC-UF: 266 ± 122x in serum, 1325 ± 376x in plasma; SEC-UC: 45 ± 25x in serum, 182 ± 79 in plasma) compared to UC alone. The technique SEC-ExSp led to a moderate increase in EV concentration from plasma samples (14 ± 12x) versus UC alone, while this EV-enrichment strategy had no benefit for isolation from serum (Figure 3b, right graph).

Simple Western immunodetection shows that the EV markers flotillin-1 and TSG101 are detectable in each of the EV samples and that they lack calnexin. Apo-A1 was still present in low amounts in EV samples purified by SEC and was also co-concentrated with UC or UF (Figure 3c and Figure S2). Compared to the concentration of Apo-A1 in the corresponding plasma, the concentration was strongly reduced in the EV samples (SEC: 37000-fold, SEC + UC: 11500-fold, SEC + UF: 3100-fold), which indicates that all of the EV-enrichment strategies are suitable to obtain sufficiently pure EVs. This was also confirmed by transmission electron microscopy (TEM), which depicts the typical EV structures for all of the EV preparation methods (Figure 3d).

### 2.3. EV Surface Protein Detection Differs Based on Isolation Methods

**Figure 4:**
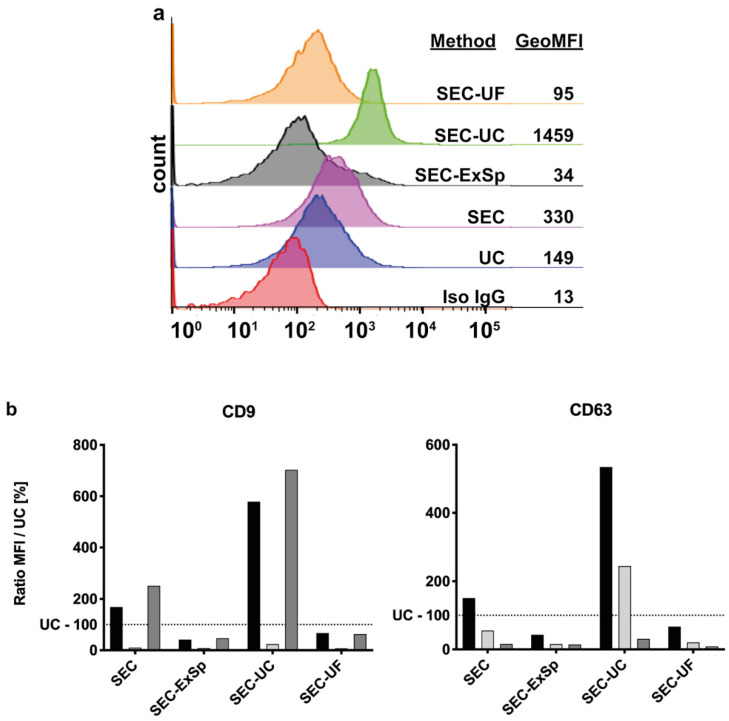
EV surface proteins differ based on the EV-enrichment strategy. (**a**) Example of bead-based flow cytometric analysis of EV surface marker CD9 according to EV separation method. The histogram shows the fluorescence intensity detected in the PE channel, including isotype control. The table indicates the geometric mean of fluorescence intensity. (**b**) Ratio of mean fluorescence intensity for standard EV markers CD9 and CD63 per particle for each EV separation technique and concentration method normalized to the value from UC (S1, S2, and S3 represent the three different donors). Note that SEC + UC led to the highest fluorescence signals with a trend towards significance (two-way ANOVA: *p* = 0.16; reference: SEC).

Since we already found differences for surface protein detection between the UC and SEC isolation methods (Figure 2e), we also assessed the impact of the different EV-enrichment strategies following SEC with respect to this. Thus, we performed bead-based flow cytometric analysis for the standard EV markers CD9 and CD63. Interestingly, we observed large differences in the signal intensity, with increased signals compared to the UC-EVs, when using UC as a concentration method after SEC, yet not when applying UF or ExSp (Figure 4a,b). Notably, we saw considerable differences between EVs from different healthy donors, indicating that there is a high EV heterogeneity between individuals.

### 2.4. Variable Protein Detection in EV Isolated by Different Methods

**Figure 5:**
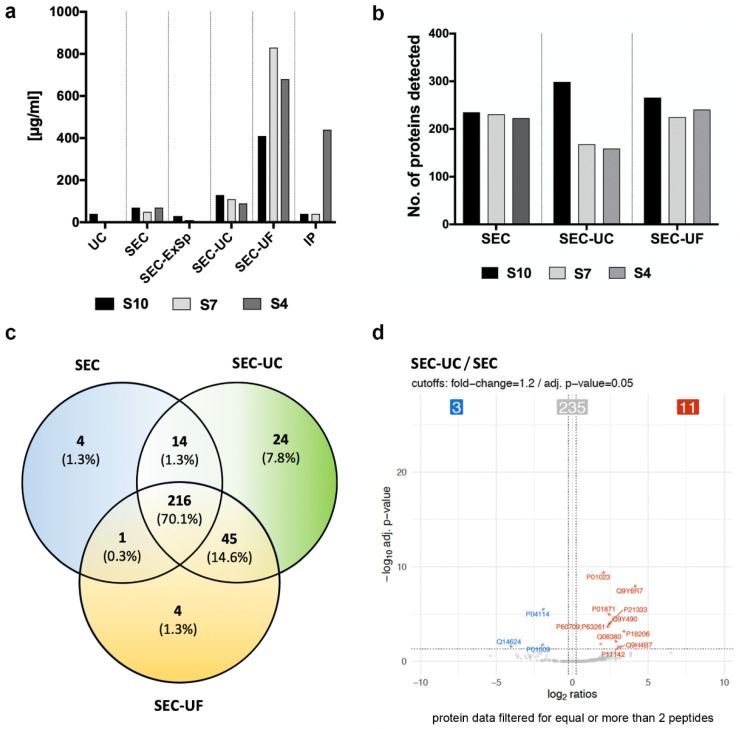
Variable protein detection depending on the EV isolation method (**a**,**b**) Differences in protein concentration and protein detection of EVs separated by different methods from human serum of three healthy volunteers (S10, S7, and S4). The highest protein concentration was obtained with SEC-UF (two-way ANOVA: *p* = 0.0002, reference: UC). (**c**) Representative Venn diagram (from volunteer S10) of proteins detected with SEC alone or when followed by concentration with UC and UF. Note the high number of shared proteins (*n* = 216, 70.6%) and that 24.7% of the proteins were detectable only after concentrating the EV sample with either UC or UF. (**d**) Significant, differentially identified proteins with SEC and SEC-UC. When applying filters of q < 0.05 and a fold-change of at least 1.2 (shown with dotted lines), only 14/256 proteins were differentially identified. Out of these, only 9 proteins showed an up- or down-regulation in at least 2/3 samples (see Table 1A). In total, 14 proteins were detected at different levels in SEC and SEC-UC, out of which *n* = 11 differed significantly in at least 2/3 donors (*n* = 6 increased and *n* = 3 decreased in SEC-UC compared to SEC). The six significantly increased proteins in SEC-UC vs. SEC were mainly Ig-related or structural proteins. In SEC-UF, three apolipoproteins were significantly reduced compared to SEC alone in at least 2/3 donors (Table 1).

Next, we performed mass spectrometric analysis of EVs enriched by each strategy to determine possible quantitative or qualitative differences in protein content. The yield of EVs from UC alone and SEC-ExSp proved to be too low for detection by mass spectrometry (Figure 5a), and no EV related proteins could be identified. The highest number of proteins was identified with SEC-UF, followed by SEC-UC and SEC; however, the numbers of proteins detected did not differ significantly (Figure 5b). As expected, most of the detected proteins were identical in all EV groups, with SEC, SEC-UC, and SEC-UF showing the most overlap (~70%). The concentration of SEC-EV with UC or UF resulted in a higher sensitivity, which was reflected in the detection of ~15% more proteins (Figure 5c,d and Figure S3).

### 2.5. Let 7a-5p, miR-23a-3p, and miR-199a-3p Are Enriched in EVs of Serum and Plasma Irrespective of Separation Method, as Opposed to miR-92b-3p or miR-128-3p

**Figure 6:**
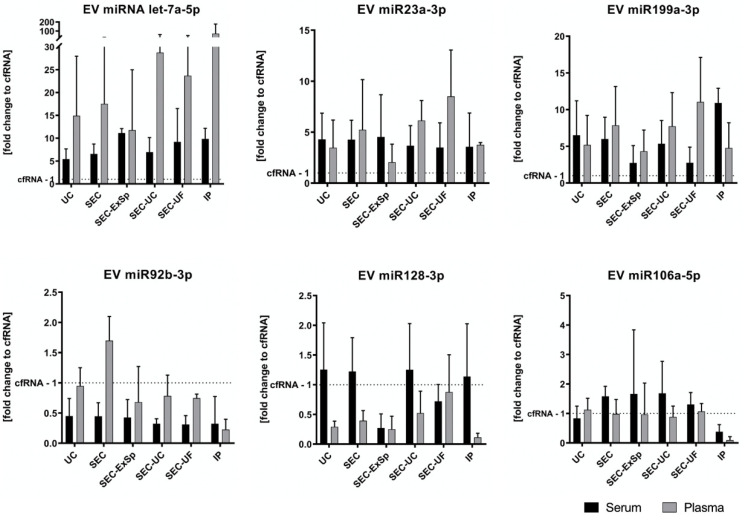
miRNA expression levels in EVs according to the different separation and concentration methods. Depicted is the fold-change of each miRNA compared to the total RNA from cell-free serum/plasma of three healthy volunteers. No significant differences between the EV separation methods were observed, but there was a high and significant variability between individuals for 4 out of 6 miRNAs (let-7a-5p: *p* = 0.002, miR-92b-3p: *p* = 0.01, miR-199a-3p: *p* = 0.01, and miR-128-3p: *p* = 0.001; two-way ANOVA). Note that let-7a-5p, miR-23a-3p, and miR-199a-3p consistently showed higher levels in EVs compared to the corresponding cell-free samples, as opposed to miR-92b-3p and miR-128-3p.

To analyze the qualitative impact of the different EV-enrichment strategies, we analyzed six miRNAs listed in the exRNA Atlas [https://exrna-atlas.org (18 April 2018)] [27]. The total cell-free material from the same serum/plasma samples was used as a reference. For qRT-PCR, reference miRNAs were chosen after multiple testing of previously described molecules (data not shown) [28]. When comparing different miRNAs, we found an enrichment of let-7a-5p, miR-23a-3p, and miR-199a-3p in EVs compared to cell-free RNA, while miR-92b-3p and miR-128-3p appeared to be EV-independent in human serum/plasma (Figure 6 and Figure S4b). miR-106a-5p had similar levels in both EVs and cell-free serum/plasma. Interestingly, PCR failed to amplify cDNA from the SEC-ExSp samples in one out of three subjects of our study (S3). While miRNAs exhibited higher levels in plasma compared to serum with cell-free RNA as a reference, no significant differences between the EV separation methods were observed. Intriguingly, significant differences between individuals irrespective of the separation technique used were detected for 4/6 miRNAs (let-7a-5p: *p* = 0.002, miR-92b-3p: *p* = 0.01, miR-199a-3p: *p* = 0.01, and miR-128-3p: *p* = 0.001; two-way ANOVA) pointing towards a heterogeneous distribution of nucleic acids across healthy volunteers.

### 2.6. Most Efficient EV-Mediated Cargo Delivery after SEC-UC Isolation

**Figure 7:**
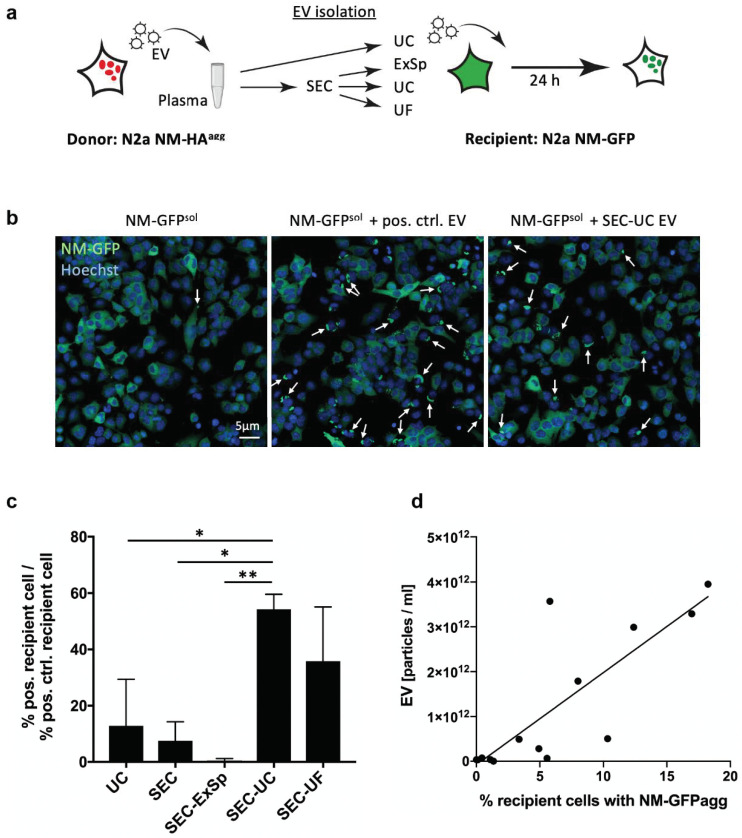
Analysis of isolation-method-dependent differences in EV function (**a**) Experimental design. EVs containing small NM aggregates from a donor neuroblastoma cell line producing NM-HA aggregates (N2a NM-HA^agg^) were collected and spiked into human plasma. EVs isolated by different methods adjusted to similar volumes were subsequently added to recipient reporter cell line expressing soluble NM-GFP (N2a NM-GFP^sol^). Uptake of EVs from donor cells results in induction of NM-GFP aggregation (NM-GFP^agg^), assessed by automated confocal microscopy. (**b**) Example Z stack images of recipient reporter cell line before (left) and after exposure to donor EVs (middle) or spiked EV isolated by SEC-UV (right). Nuclei were stained with Hoechst. White arrows indicate NM-GFP aggregate formation. (**c**) Reporter NM-GFP^agg^ induced by exposure of recipient N2a NM-GFP^sol^ cells to spiked EVs. Shown are the percentages of recipient cells with induced NM-GFP^agg^ 24 h post exposure to EVs. Aggregate induction by spiked plasma EV samples relative to induction by non-spiked UC-EVs from donor cells is shown. SEC + UC EVs isolation resulted in highest percentage of cells with NM-GFP^agg^ (* *p* < 0.05, ** *p* < 0.01, two-way ANOVA; *n* = 3 independent experiments). (**d**) Spearman correlation of NM-GFP aggregate formation and concentration of all EVs irrespective of the isolation method. A high correlation between parameters was found (Spearman r = 0.85 (95% CI: 0.561–0.952), *p* = 0.0003).

To test whether the isolated EVs were functional with respect to cargo delivery, we made use of our recently established murine cell assay that relies on the induction of reporter protein aggregates in recipient cells upon exposure to EV derived from donor cells propagating protein aggregates composed of the prion-like domain NM of *S. cerevisiae* Sup35 [29]. Recipient cells normally express soluble GFP-tagged reporter protein NM (NM-GFP^sol^). Upon exposure to NM aggregate-loaded EV, donor aggregates gain access to the cytosol, where they act as seeds that induce aggregation of the reporter. Induction of NM-GFP aggregates (NM-GFP^agg^) thus demonstrates functionality of EV fractions [29]. Interestingly, aggregate induction efficiency of SEC-UC-EV (measured by cells with NM-GFP^agg^) was significantly higher (54 ± 4% of pos. ctrl, *p* = 0.015 with SEC as a reference) than of EVs isolated by all other methods, except for SEC-UF (35 ± 16% of pos. ctrl; *p* = 0.153 with SEC as a reference, Figure 7c). The latter showed a non-significant trend towards higher efficacy in NM-GFP aggregate formation compared to EVs from SEC, UC, and SEC-ExSp (Figure 7c). A comparison to NM-GFP aggregate formation induced by the positive control (not spiked into samples) reveals that the spiked-in EVs could not completely recover the activity of EVs isolated from conditioned medium (indicated as 100%, Figure 7). Again, SEC followed by precipitation (SEC-ExSp) was the least effective strategy (0.5 ± 0.5% of pos. ctrl). We found a high correlation between NM-GFP aggregate formation and EV particle concentration (Spearman r = 0.85; *p* = 0.0003) (Figure 7b). EVs from the plasma of healthy volunteers co-isolated with the applied EV isolation strategies had no impact on spontaneous NM-GFP aggregate formation (data not shown). Thus, except for SEC-ExSp, we were able to recover functional EVs with all procedures, albeit to different degrees.

## 3. Discussion

EVs have been recognized to contain potential biomarkers for diagnosis and monitoring of disease progression or treatment response in a wide range of diseases. Nevertheless, EVs are not yet established for routine diagnostics. This is partly due to the fact that studies use different EV separation protocols and therefore the results cannot be directly compared. The aim of this study was to identify the EV separation method that is most suitable and reproducible for biomarker analysis from small amounts of serum and plasma, including both proteins and nucleic acids. This method should also be compatible with routine diagnostic laboratories procedures. In this study, we compared different EV isolation protocols with regard to quantitative and qualitative protein yield, as well as the nucleic acid contents, and we have come to a conclusion about the best method to use.

By comparing the major separation approaches used, namely ultracentrifugation (UC), size exclusion chromatography (SEC), and immunoaffinity isolation (IP), we found that (i) all methods are suitable to isolate EVs; (ii) the yield of EVs differs considerably between the different methods; (iii) unexpectedly, the methods showed little differences in miRNA profile; (iv) UC-EVs were best suited for flow cytometric analysis; and (v) EVs isolated by IP are unsuitable for further protein analysis. In order to check whether the concentration of EVs obtained via SEC improves the subsequent analyses, we applied ultracentrifugation (SEC-UC), ultrafiltration (SEC-UF), or precipitation of SEC-EVs using Exo-spin^TM^ exosome purification buffer (SEC-ExSp). Our results indicate that (a) for all three methods, the sensitivity of miRNA detection had no benefit from concentration; (b) for the SEC-UC and SEC-UF methods, there was an increase in protein detection by using proteomics; (c) for SEC-UF concentrated EV, there was a loss of flow cytometry signal; (d) for the SEC-ExSp method, it was found to be inefficient and caused a substantial loss of particles, resulting in reduced signal strength in both protein and nucleic acid analyses; and (e) SEC-UC- and SEC-UF-isolated EVs showed the greatest capacity for cargo delivery in a functional assay. Based on these findings, we have concluded that SEC-UC, is the most suitable and consistent method for EV separation for both serum and plasma samples.

Characterization of EVs with respect to size, purity, and morphology showed that all of the applied EV-enrichment strategies (UC and SEC, optionally followed by further concentration strategies, or immunoprecipitation) resulted in pure small EVs of the expected size (100–160 nm). All of the isolated EVs contained typical EV markers, such as flotillin-1 and TSG101 [30], and lacked calnexin, whose presence would indicate contamination with endoplasmic reticulum-derived particles. The yield of EVs from SEC was significantly higher compared to those obtained by UC. This might in part be due to the higher abundance of lipoproteins such as Apo-A1, which were less effectively removed by SEC compared to UC or IP. Nanoparticle tracking analysis cannot distinguish between EVs and lipoproteins. Thus, the higher yield observed in EV from SEC does not necessarily reflect a greater number of EVs. In a direct comparison between EVs isolated using UC and different fractions of the SEC eluate, we found the highest levels of surface antigens on UC-EVs. This indicates that there is more contamination by non-EV proteins in the SEC samples compared to UC. Similar findings were reported by Takov and colleagues, confirming the purity of EV separated by UC [26]. This loss of signal strength per particle through SEC purification might cause loss of detection of low abundance surface proteins. However, differential UC is time-consuming and thus not suitable for laboratories of routine diagnostics. Some further disadvantages of UC, according to MISEV2018, are the low specificity of the method and the inter-laboratory differences seen due to different rotor types [13].

While SEC would then seem a more appropriate method for a diagnostic laboratory, SEC alone needs a subsequent concentration method since the purified EVs are dispersed over several fractions. As an example, isolation of EV from 500 µL of serum/plasma results in a final volume of 1.5 mL when the EV-containing fractions 8–10 are pooled. Hence, we studied different methods for concentrating the EV after SEC. Interestingly, subsequent concentration of SEC-EVs by ultrafiltration did not lead to an overall signal increase in the surface proteins CD9 and CD63, which are two standard surface markers of small EVs [31], although particle concentration and thus particle number used for bead-based flow cytometry was much higher. One possible explanation for this finding could be that the accessibility of the surface proteins might be impaired by a condensed corona induced by the increased concentration [32]. Additionally, the comparatively reduced signal strength might result from competitive binding of non-EV proteins, mainly lipoproteins, to the polystyrene beads since they are co-concentrated, as shown for Apo-A1. The number of contaminating lipoproteins after SEC could possibly be reduced if a membrane with a cut-off of 100 kDa was used. Consecutive ultracentrifugation of SEC-EV increased signal strength compared to all the other concentration techniques. Since the EV suspension after SEC (1.5 mL) has a much lower volume compared to the necessary a priori dilution of plasma/serum samples when UC alone is to be performed (e.g., 500 µL plasma/serum diluted to 11 mL), EV pelleting can be performed in 1.5 mL reaction tubes. The use of a smaller rotor with up to 12 slots also allows parallel processing of more samples, which is of high importance for routine laboratories. Interestingly, a combination of these methods has been proposed by Koh et al., however in the opposite order [21]. Nevertheless, initially using SEC to avoid co-isolation of further particles and omitting large-volume UC as a first-step would favor conducting SEC prior to UC, as performed in this study.

SEC-ExSp showed by far the lowest signals for CD9 and CD63. This is in line with the extremely low recovery of SEC-EV by precipitation with Exo-spin^TM^, clearly illustrating that this enrichment strategy is of no benefit compared to SEC alone or UC. One could presume that a higher protein concentration than is present in SEC-isolated EVs is needed for successful precipitation of the EVs. This suggests that precipitated EVs are contaminated by other (non-EV) proteins, resulting in poorer purity. Concerns about the purity and functionality of EVs isolated by precipitation have been frequently expressed [13,33]. Indeed, in this study, we do not only see lower signals for surface proteins, but also highly compromised EV function, as mentioned below.

Apart from SEC-based techniques and UC, we also included immunoprecipitation in our study. Several IP-based protocols using magnetic beads coated with antibodies against standard EV markers CD9, CD63, and CD81 [23,34] or tumor-associated antigens, such as EpCAM, EGFR, or CD44 [24,25,35], have been reported. System Biosciences introduced ExoQuick enrichment of EVs, followed by IP capture with larger (9.1 µm) magnetic beads modified with streptavidin and biotinylated antibodies against CD9, CD63, and CD81 for biofluids. While the bead size and the covalent streptavidin–biotin reaction has advantages over other methods, the initial precipitation step harbors the already discussed drawbacks. The IP method used in this study was developed by Miltenyi Biotec [23] and is capable of separating EVs with an unequal distribution of the three standard markers (e.g., EVs from B and NK cells being devoid of CD9 and from platelets being devoid of CD81 [23,36]) and could thus comprise a reliable method for biomarker studies. Notably, however, a disadvantage of this method is that EV quantification with NTA is not easily done, as magnetic beads and EVs fall within the same size range. At the same time, IP-isolated EVs are incompatible with flow cytometric analysis for several reasons. Again, due to the small size of the MicroBeads, the addition of larger beads is required (for instance, unspecific protein-binding polystyrene beads) since most flow cytometry instruments do not meet the resolution requirements for particles smaller than ~250 nm. Moreover, a primary–secondary antibody-based staining is impossible because of the concomitant detection of the capture antibodies, which leads to a strong background signal. Staining with directly labeled antibodies results in decreased signal strength, which could impair detection of weakly expressed proteins. Moreover, studies of the function of IP-isolated EVs are limited owing to potential interference of the beads and antibodies with certain assays. One future direction would be the development of a method to efficiently detach the beads from the EVs by releasing the bond between antibody and antigen. Commercially available IgG elution buffer allows the user to break antigen–antibody interactions but unfortunately also releases antibody from the beads, thus resulting in high background in flow cytometry. Hopefully soon, antigen-releasing antibodies that have already been developed for label-free isolation of cells (e.g., REAlease releasable antibodies, Miltenyi Biotec) can be adapted to EV isolation technology. IP would offer an ideal EV separation method for serum/plasma with the use of disease-specific antigens. Such an attempt has already been employed for glioblastoma microvesicles, using a magnetic microfluidic system [12]. This is crucial since physiological EVs from healthy volunteers and disease-EVs from patients show big differences in terms of their cargo and content [10,25]. Future studies are warranted to define the appropriate antigens for different diseases and thereby allow disease-specific EVs selection for biomarker analysis.

The use of EV proteins as biomarkers for disease progression has already been described [10]. However, proteomic analysis of EVs isolated from human serum or plasma offers a technical challenge, owing to low protein yield and contamination with lipoproteins and other abundant serum components. Indeed, EV yield and recovery proved to be essential for successful proteomic analysis, as no EV-related proteins were identified with UC-EVs and SEC-ExSp-EVs, the samples with the lowest protein concentration in our study. Smolarz and colleagues have recently shown that the discovery of vesicle-specific proteins is feasible with SEC-derived EVs from small amounts of human serum [37]. This supports our findings that SEC or SEC followed by UF or UC results in the highest protein content. Notably, the differentially identified proteins between SEC and SEC-UC or SEC-UF EVs (mainly Ig-related or cytoskeleton proteins) did not point towards functionally different EVs, arguing that these concentration methods after SEC do not drastically change the composition of EV proteins.

We observed high heterogeneity between donors for several different miRNAs, which indicates the absence of a “global” miRNA profile of healthy volunteers. While we did not see any significant differences according to the purification method, we saw a slight tendency towards higher Ct values for SEC-UC-EV and detected clear miRNA-dependent differences in EV enrichment. SEC-ExSp purification seemed to inhibit PCR in one out of three subjects, which underlines a further drawback of precipitation-based EV separation techniques [38]. Let-7a-5p, as well as miR-23a-3p and miR-199a-3p copies, were much higher in EV samples compared to cell-free serum/plasma; miR106a-5p exhibited slightly higher levels in EV, and miR-92-3p as well as miR-128-3p appeared to not be enriched in EV. Let-7a-5p has previously been described to be selectively packaged into exosomes in vitro and in vivo [27,39]. Similar results of miRNA upregulation in EV have been reported for miR-23a-3p in rat plasma [40]. In contrast, miRNAs such as miR-16 or miR-24 were reported to associate with ribonucleoproteins and not EV [41]. Our data might point to a similar EV-independent mechanism for the release of miR-92-3p and miR-128-3p, but this remains to be confirmed. Concerning biomarker studies, our results indicate that, based on the miRNA expression pattern, a choice between (a) RNA analysis after EV separation from blood or (b) total cell-free RNA analysis is necessary.

To assess possible functional differences, we utilized a reporter system introduced by Vorberg et al. [42] that relies on the detection of reporter protein aggregates upon exposure of recipient cells to EVs derived from donor cells producing protein aggregates of the same kind. In this system, a murine donor cells line stably producing aggregates composed of the ectopically expressed prion domain NM of the *S. cerevisiae* protein Sup35 releases EVs that contain small NM oligomers. Upon addition of these EVs to recipient cells expressing the same protein in its soluble form, EVs mediate the uptake of these protein seeds into the cytosol, where the protein aggregates initiate aggregation of the GFP-tagged reporter NM [43,44,45]. The formation of GFP-tagged NM aggregates as soon as 6 h post EV exposure serves as a read-out for functional EV delivery of active cargo to recipient cells [29]. The dependence of this read-out on EVs has been confirmed by the colocalization of the protein aggregates with classical EV markers (flotillin-1, ALIX, TSG101) [46]. We demonstrated a clear superiority of SEC-UC and to a lesser extent SEC-UF over all of the other separation methods (Figure 7). Notably, SEC-ExSp again led to the lowest yield of functional EVs. Consistent with results from Mol et al. [47] and excluding SEC-ExSp for all the previously mentioned reasons, all SEC-based techniques were superior to UC, which implies that SEC should be the method of choice for functional analyses of EVs from biological material. Moreover, the high correlation of particle numbers and recipient cells with induced reporter aggregates argues that primarily the particle concentration, and not qualitative differences, is responsible for the functional readout.

Here we studied both serum and plasma in most experiments. The discussion on suitability of the two biological materials for EV experiments is long and ongoing [48]. So far, most EV studies have been conducted with plasma samples. Yet, EV isolation protocols have also been developed for serum and can be applied to most biofluids according to the recent MISEV position paper [13]. Large-scale studies demonstrated that analyzed miRNAs did not differ between serum and plasma, but this was attributed to the RNA isolation methods [27]. While differences in protein content have not been studied using different EV isolation protocols, there is consensus that both biofluids are suitable for protein analysis, as long as all experimental procedures (EV separation, concentration methods, RNA and protein extraction methods) are reported accurately based on MISEV guidelines [13]. Notably, preanalytical variables such as sample handling and vesicle preparation play a major role in downstream analyses [4,49]; therefore, their documentation is of utmost importance not only in trial and publication reports but also in future standard operating procedure (SOP) for a clinical setting.

Taken together, with this comprehensive study, we identified method-related differences in yield, purity, and downstream analyses for EV, especially regarding EV protein analysis. To our knowledge, this is the first report of such an integrated approach that assesses protein and RNA content of EV from both serum and plasma. We hereby conclude that due to the positive effects on particle yield, EV proteins and the negligible differences in miRNAs, the most appropriate method for biomarker studies is SEC followed by concentration with UC or possibly UF. A more specific approach to isolate EV using IP that targets disease-specific antigens could be a promising technique. Its feasibility is yet to be determined in future studies.

## 4. Materials and Methods

### 4.1. Ethics Approval

Samples were obtained as approved by the ethics committees of the University Hospital of Bonn (AZ 007/17). All donors gave written and informed consent.

### 4.2. Sample Collection

Blood was drawn from healthy volunteers by venipuncture, using 21G needles (Safety-Multifly-Needle, Sarstedt, Nuembrecht, Germany) and collected in 9 mL serum (S-Monovette, Sarstedt, Nuembrecht, Germany) or plasma (EDTA KE Monovette, Sarstedt, Nuembrecht, Germany) tubes. Serum tubes were stored in an upright position for 20 min to allow clotting of blood and subsequently centrifuged for 15 min at 2000× *g* at room temperature (RT). Plasma tubes were centrifuged within 30 min after the blood was drawn at 1200× *g* for 20 min at RT. After the first centrifugation step, both plasma and serum samples were transferred to a fresh 15 mL tube and centrifuged again for 15 (plasma) or 20 (serum) min at 3200× *g* at 6 °C to ensure complete removal of platelets. After the second centrifugation step, the supernatants were filtered with a 0.45 µm filter and stored in aliquots of 0.5 mL at −80 °C.

### 4.3. Isolation of EVs

EVs were isolated from serum or plasma using differential ultracentrifugation (UC), size exclusion chromatography (SEC), or immunoprecipitation (IP).

#### 4.3.1. Purification of EVs by Differential Ultracentrifugation (UC)

After the pre-preparation of serum or plasma (described above), 0.5 mL aliquots were diluted ~1:20 in ice-cold Hanks Balanced Salt Solution (HBSS) and large EVs and microparticles were removed by centrifugation at 10,000× *g* for 45 min. The pre-cleared supernatant was then subjected to UC at 100,000× *g* for 1 h 45 min to sediment the small EVs. The EV pellet was resuspended in 10 mL HBSS followed by UC at 100,000× *g* for 1 h 45 min to wash and pellet the small EVs. The final pellet was resuspended in 120 µL HBSS + protease inhibitor (Roche, Basel, Switzerland) (HBSS + PI). All UC steps were performed in a SW41 Ti Swinging Bucket rotor (*k* factor of 204, Beckman Coulter, Brea, CA, USA) at 4 °C.

#### 4.3.2. Purification of EVs by Immunoprecipitation (IP)

The 0.5 mL samples of serum and plasma were diluted with an equal volume of HBSS and centrifuged at 10,000× *g* for 45 min. Subsequently, EVs were captured using the Exosome Isolation Kit Pan (MicroBeads conjugated to CD9, CD63, and CD81) with µColumns and µMACS separator, according to the manufacturer’s protocol (all Miltenyi Biotec, Bergisch Gladbach, Germany). EVs were eluted in 120 µL HBSS + PI.

#### 4.3.3. Isolation of EV by Size Exclusion Chromatography (SEC)

EVs from plasma and serum samples were isolated using the sepharose-based qEV columns (iZON Science, Christchurch, New Zealand). The samples were thawed after freezing and 0.5 mL plasma or serum was applied to the column. The EVs were eluted with HBSS. Fractions of 500 µL each were collected and fractions 8–12 were used for further analysis.

### 4.4. Concentration of EVs after Isolation by SEC

For comparison of different concentration methods, fractions 8–10 from 1.5 mL plasma (3 × 0.5 mL runs) were pooled, resulting in a total volume of 4.5 mL eluate. Protease inhibitor was added to a final concentration of 1×. Subsequently, 1.45 mL of this mixture were concentrated to a final volume of 120 µL by (a) ultracentrifugation (SEC-UC; 1 h 45 min, 110,000× *g*, 4 °C) using the TLA-55 rotor and the Optima MAX-XP ultracentrifuge (both Beckman Coulter, Brea, CA, USA); (b) ultrafiltration (SEC-UF) using Amicon Ultra-2 Centrifugal Filter devices with a cut-off of 10 kDa NMWL (Merck Millipore, Burlington, MA, USA; SEC-UF: concentration of sample at 4000× *g*, RT for 20 min and elution at 1000× *g*, RT for 2 min); or (c) precipitation (SEC-ExSp) with an Exo-spin^TM^ exosome purification kit (Cell guidance systems, Cambridge, UK) according to the manufacturer’s instructions. Briefly, the EV-containing solution was mixed with Exo-spin buffer at a 2:1 ratio and was incubated at 4 °C for 60 min, followed by a 1 h centrifugation at 16,000× *g* and 4 °C. For SEC and elution, Exo-spin columns were used according to the manufacturer’s instructions. All EVs were eluted in 120 µL HBSS + PI.

### 4.5. Quantification and Characterization of EVs

#### 4.5.1. Nanoparticle Tracking Analysis (NTA)

For NTA, ZetaView Nanoparticle Tracking (Particle Metrix, Meerbusch, Germany) was used according to the manufacturer’s protocol, as previously described [50]. EV samples were diluted in HBSS to a final volume of 1 mL (dilution range: 1:50–1:3000). Ideal measurement concentrations were found by testing the appropriate particle per frame number (140–200 particles/frame). The manufacturer’s default software settings for EVs were used. For each measurement, two cycles were performed by scanning 11 cell positions each and capturing 30 frames per position under the following settings: focus: autofocus; camera sensitivity for all samples: 79; shutter: 70; scattering intensity: detected automatically; cell temperature: 23 °C. After capture, the videos were analyzed by the built-in ZetaView Software 8.05.11 SP1 with specific analysis parameters: maximum area: 1000, minimum area: 5, minimum brightness: 30. Hardware: embedded laser (40 mW at 488 nm); camera (CMOS).

Isolated PAN-EV microbeads were pelleted at 20,000× *g* for 45 min and the supernatant was discarded. The microbeads were then resuspended in 220 µL IgG elution buffer (Thermo Fisher Scientific; Waltham, MA, USA) and incubated for 30 min at 23 °C with 450 rpm (Eppendorf ThermoMixer shaker; Eppendorf, Hamburg, Germany) to disrupt antibody–antigen binding. The microbeads were pelleted again (same centrifugation conditions as before), and the supernatant was used for NTA and TEM analysis.

#### 4.5.2. Flow Cytometry Assays

Bead-assisted flow cytometry: 100 µL of each EV suspension isolated by UC or SEC were incubated with 4 µL carboxylated polystyrene beads (4.42 µm, 5 × 10^7^ beads/mL; Polysciences, Warrington, PA, USA) overnight at 4 °C on rotation. The concentration of the beads was chosen to be as low as possible and still be able to acquire enough events for a valid statistical analysis (5000). Next, the beads were blocked by adding an equivalent volume of PBS supplemented with 2% BSA and incubated for 1 h at RT, shaking at 800 rpm. Bead-coupled EVs were split into wells and primary antibodies (anti-CD9: clone HI9a, anti-CD63: clone H5C6, iso-IgG: clone MOPC-21; all BioLegend, San Diego, CA, USA) were added to a final dilution of 1:100. After 20 min incubation at RT, the beads were pelleted by centrifugation at 800× *g* for 3 min and washed twice with 200 µL PBS (containing 1% BSA, 0.1% sodium azide) and then stained with 1:200 diluted PE-labeled secondary antibody (goat-anti-mouse: clone Poly4053; BioLegend) for 20 min at RT. The washing steps were repeated, and the beads were analyzed immediately. Gating of beads was performed based on FSC/SSC parameters, which allowed for the exclusion of possible unbound EV-antibody aggregates as well as bead-doublets from the analysis. Data were acquired with LSRII or FACSCanto (both BD Biosciences, San Jose, CA, USA) and analyzed with FlowJo software, version 10 (BD Biosciences). The geometric mean fluorescence intensities were background-corrected and negative values were excluded from the plot.

MACSPlex analysis: Bead-based analysis using MACSPlex exosome capture beads was performed according to the kit guidelines [23]. Briefly, 15 µL of beads were incubated with 120 µL EV suspension overnight at RT. After a washing step with 500 µL MACSPlex buffer (centrifugation at 3000× *g* for 5 min), 15 µL of MACSPlex Exosome detection reagent cocktail (CD9-, CD63-, CD81-APC) were added and incubated for 1 h at RT. After two additional washing steps with MACSPlex buffer, the pellet was resuspended in 150 µL and analyzed, as described above. After gating the bead population based on FSC/SSC parameters, as previously mentioned, different gates in the PE versus FITC channel for 37 different antigens were applied.

#### 4.5.3. Transmission Electron Microscopy (TEM)

The established protocol of Bachurski et al. [50] was applied. Briefly, 5 µL of each sample was loaded onto formvar-coated copper grids (Science Services, Munich, Germany). After 20 min incubation, the grid-bound EVs were fixed with 2% paraformaldehyde for 5 min. Samples were washed with PBS and fixed again with 1% glutaraldehyde for 5 min, washed with Milli-Q water, and incubated with contrast dye (1.5% uranyl acetate) for 4 min. Images were acquired using a Gatan OneView 4 K camera (Gatan, Pleasanton, CA, USA) mounted on a Jem-2100Plus microscope (JEOL) operating at 200 kV.

### 4.6. Immunodetection of EV Proteins

Presence of the EV markers TSG101 and flotillin-1, the endoplasmic-reticulum protein calnexin and apolipoprotein A1 (Apo-A1) was analyzed by protein separation and immunodetection using Simple Western technology with the Wes instrument (ProteinSimple, San Jose, CA, USA). The 12-230 kDa Wes Separation Module as well as the secondary anti-rabbit, anti-mouse, and anti-goat antibody detection modules (all ProteinSimple) were used according to the manufacturer’s instructions. In short, for each well 3 µL EVs were diluted with 1 µL 0.1× sample buffer and 1 µL 5× fluorescent master mix. Calnexin (clone: C5C9; Cell Signaling Technology, Danvers, MA, USA) and TSG101 (polyclonal; abcam, Cambridge, UK) were probed in one capillary and detected with HRP-conjugated anti-rabbit secondary antibody. Anti-flotillin-1 (clone 18/flotillin-1; BD Biosciences, CA, USA) binding was detected with HRP-conjugated anti-mouse secondary antibody; anti-Apo-A1 (polyclonal, R&D Systems, Minneapolis, MN, USA) binding was detected with HRP-conjugated anti-goat secondary antibody. The default run conditions were changed to 22 s stocking gel uptake, 15 s sample uptake, and 90 min primary- and 40 min secondary-antibody incubation. All other settings were left on the default setting. Analysis was performed with Compass software (ProteinSimple, San Jose, CA, USA).

### 4.7. Proteomic Analysis

Vesicles in HBBS containing protease inhibitor were subjected to disruption in five freezing (liquid N_2_) and thawing (30 °C, 3 min, 1200 rpm in an Eppendorf ThermoMixer shaker, Eppendorf, Hamburg, Germany) cycles followed by incubation for 5 min in an ultrasonication bath (Sonorex, BANDELIN electronic GmbH & Co. KG, Berlin, Germany) at RT. After centrifugation (60 min, 13,000× *g*, 4 °C), the supernatant containing soluble proteins was transferred into a new vial and the protein concentration was determined using a Bradford assay (Biorad, Munich, Germany); 2 µg of each sample were reduced with 10 µL 25 mM dithiothreithol in 20 mM aqueous ammonium bicarbonate buffer (ABC-buffer) for 30 min at 30 °C and subsequently alkylated with 10 µL 100 mM iodoacetamide in 20 mM ABC-buffer for 15 min at 30 °C. Then, 3 µL of SP3 beads [(hydrophobic: Sera-Mag Speedbeads carboxylate-modified particles (Thermo Fisher Scientific, Waltham, MA, USA); hydrophilic: Speedbead magnetic carboxylate-modified particles (GE Healthcare, Chicago, IL, USA)] were added to each sample and the solution was adjusted to 70% (*v/v*) acetonitrile: water. Subsequently, the SP3-protocol for protein digestion using trypsin and peptide purification was performed as described previously [51]. Peptides were resuspended in 2% (*v/v*) dimethyl sulfoxide and mixed with equal amounts of doubly concentrated LC buffer (4% (*v/v*) acetonitrile, 0.2% (*v/v*), and 1/50 HRM peptides prepared according to the manufacturer’s instructions (BiognoSYS, Schlieren, Switzerland)).

Peptides were separated in an UltiMate 3000 nano-LC system (Thermo Fisher Scientific, MA, USA) using a binary gradient of buffer A [0.1% (*v/v*) acetic acid in HPLC-grade water (Thermo Fischer Scientific, MA, USA)] and buffer B [0.1% (*v/v*) acetic acid in ACN] at a flow rate of 300 nL/min Subsequently, peptides were analyzed with a Q Exactive^TM^ Plus mass spectrometer (Thermo Fisher Scientific, Waltham, MA, USA) in data independent acquisition (DIA) [46]. Details are provided in the Supporting Information Table S1.

Raw data were analyzed using Spectronaut version 13.10 (BiognoSYS, Schlieren, Switzerland) in direct DIA mode against a Uniprot database limited to human entries (04/2019, 20,416 entries). For theoretical digestion, trypsin/P was set as enzyme with up to two missed cleavages allowed. Oxidation of methionine was set as variable and carbamidomethylation of cysteine as fixed modification. IRT peptide profiling was enabled for non-identified profiling as well as a global sparse imputation based on a protein q-value cut-off of 0.01.

### 4.8. RNA Extraction from EV and Total Cell-Free Serum/Plasma and Quantitative Real-Time PCR (qRT-PCR)

For RNA extraction, the RNeasy micro-kit was used according to the manufacturer’s protocol (Qiagen, Hilden, Germany). After RNA extraction, equal volumes of the RNA samples were used for advanced cDNA synthesis using TaqMan Advanced miRNA cDNA Synthesis Kit according to the manufacturer’s protocol (ThermoFisher Scientific, Waltham, MA, USA). Briefly, a poly (A) tailing reaction was performed, followed by an adaptor ligation reaction and reverse transcription. After a non-specific preamplification step (miR-Amp Reaction), qRT-PCR was performed in QuantStudio 7 using TaqMan Advanced Control miRNA Assay (Table S2, both ThermoFisher Scientific, Waltham, MA, USA). miR-103a-3p and miR-484 were used as reference miRNAs (Table S2). The QuantStudio 7 PCR run was comprised of enzyme activation at 95 °C for 20 s, followed by 40 cycles of denaturation (1 s at 95 °C) and annealing/extension (20 s at 60 °C).

### 4.9. EV Cargo Delivery—Function Assay

EV isolation for functional analysis. A recently described cell assay was used that is based on the prion-like activity of protein aggregates derived from the *Saccharomyces cerevisiae* Sup35 NM prion domain expressed in mouse neuroblastoma cells [45]. For the production of EVs loaded with NM aggregates, the N2a subclone (N2a NM-HA^agg^ s2E) was chosen [45]. This clone continuously produces HA epitope-tagged NM aggregates and was selected for its production of EVs capable of efficiently inducing aggregation of soluble GFP-tagged NM in recipient cells (N2a NM-GFP^sol^). We have previously shown that NM aggregates are released in association with EVs [45]. Intact EVs are required for efficient NM aggregate transfer to recipient cells. For EV production, 9 × 10^6^ donor cells per T175 five-layer flask were grown in OptiMEM supplemented with 10% EV-free fetal bovine serum and antibiotics. Seventy-two hours post plating, conditioned medium was collected and the EVs were isolated with classical differential UC, as described above.

#### Reporter Assay for Functional EVs

Isolated EVs from the donor clone N2a NM-HA^agg^ were spiked into healthy plasma and EV separation was performed with five different methods (UC, SEC, SEC + ExSp, SEC + UC, and SEC + UF), as described above. Plasma spiked with an equal volume of HBSS + PI served as a negative control to determine the impact of healthy plasma EVs on spontaneous NM-GFP aggregate formation. Pure donor cell EVs were used as a positive control. To test for functional EVs, 6 × 10^3^ recipient N2a NM-GFP^sol^ cells in 30 µL per well were plated on CellCarrier-384 black microplates (PerkinElmer, Solingen, Germany). From two to four hours later, cells were exposed to EVs (10 µL) separated by different methods, as described above. Each preparation was tested in triplicate. Recipient cells were grown for 24 h and subsequently fixed by the addition of 4% paraformaldehyde. Nuclei were counterstained with 4 μg/mL Hoechst. Automated image acquisition and analyses were performed using the Yokogawa CellVoyager CV6000 confocal microscope, equipped with a 20× objective. Maximum intensity projections were generated from Z-stacks. Images were acquired from 16 random fields per well with an average of 3 × 10^3^ to 4 × 10^3^ cells/well. For object recognition and data analyses, a recently developed algorithm was used [45]. Experiments were each performed three times. Aggregate formation was calculated as a ratio normed to the positive control.

### 4.10. EV Track

We have submitted all relevant data of our experiments to the EV-TRACK knowledgebase (EV-TRACK ID: EV200005) [52]. Our EV-METRIC is 75%.

### 4.11. Statistics

For statistical analyses, GraphPad Prism (Version 8.2.1) software was used (La Jolla, CA, USA). Two-way pairwise ANOVA with Dunnett’s and Tukey’s multiple comparison test was applied to evaluate the differences between separation techniques but also between individuals (column and row factor).

For proteomic analysis, the entire DIA-MS analysis was performed using R [R version 3.5.2 (20 December 2018)]. The raw data output from Spectronaut was used to perform a median normalization over the MS2 total peak area intensities (EG.TotalQuantity) for all ions possessing a q-value < 0.01. Zero intensity values were replaced using the half-minimal intensity value from the whole dataset. To generate peptide intensity data, the sum over ions per sample and peptide was calculated. The Hi3 protein-intensity data were generated by picking common high intensity peptides (2–3 peptides) over all samples per protein by using the median and subsequently calculating the average of the picked peptides. The principal component analysis for the peptide and protein levels was done using the factomineR package (version: 1.41), in which the data were scaled to unit variance. Statistical analysis was performed in a pairwise manner using a moderate *t*-test for proteins identified with at least two peptides. Protein-intensity ratios between two conditions with an absolute fold change > 1.2 and an adjusted *p*-value ≤ 0.05 (Benjamini-Hochberg) were regarded as significantly different. All plots were generated using the factoextra package (version: 1.0.5), tidyverse package (version: 1.2.1), ggrepel package (version: 0.8.0), GGally package (version: 1.4.0), ggcorrplot package (version: 0.1.2), or plotly package (version: 4.8.0).

## 5. Conclusions

We have conducted a study of bloodstream EVs evaluating differences in both protein and RNA analyses. We conclude that the most appropriate EV separation method for biomarker studies from serum or plasma is SEC followed by concentration with UC and possibly UF. EV capturing via IP that targets disease-specific antigens is a novel promising technique, which has to be optimized and further evaluated in future studies.

## Figures and Tables

**Table 1:** Significantly differentially detected proteins upon EV concentration strategy.

**A**				
**Uniprot Accession No.**	**Gene ID**	**Description**	**SEC-UC vs. SEC**	**Mean Ratio** **SEC–UC/SEC**
Q14624	ITIH4	Inter-alpha-trypsin inhibitor heavy chain H4	**↓**	0.72
P01009	SERPINA1	Alpha-1-antitrypsin	**↓**	0.72
P04114	APOB	Apolipoprotein B-100	**↓**	0.83
P04275	VWF	von Willebrand factor	**↑**	3.19
P01023	A2M	Alpha-2-macroglobulin	**↑**	5.62
P60709; P63261	ACTB	Actin, cytoplasmic 1	**↑**	4.31
P01871	IGHM	Ig mu chain C region	**↑**	6.71
Q08380	LGALS3BP	Galectin-3-binding protein	**↑**	5.71
Q9Y6R7	FCGBP	IgGFc-binding protein	**↑**	10.19
**B**				
**Uniprot Accession No.**	**Gene ID**	**Description**	**SEC-UF vs. SEC**	**Mean Ratio** **SEC–UF/SEC**
P02647	APOA1	Apolipoprotein A-I	**↓**	0.27
P06727	APOA4	Apolipoprotein A-IV	**↓**	0.11
P02649	APOE	Apolipoprotein E	**↓**	0.14

Differentially identified proteins in (**A**) SEC-UC compared to SEC and (**B**) SEC-UF compared to SEC, with filters of q ˂ 0.05 and a fold-change of at least 1.2. Note the low overall number of proteins with differential detection in total and that only SEC-UC led to increases in protein detection (green arrows, 6 proteins). *n* = 3.

## Data Availability

The datasets generated during and/or analyzed during the current study are available from the corresponding author on reasonable request. Proteomics data are included in the Supplementary Table S1.

## Supplementary Materials

The following are available online at https://www.mdpi.com/article/10.3390/ijms22179211/s1.

## Abbreviations

EVs	extracellular vesicles
ExSp	precipitation using ExoSpin
HBSS	hanks balanced salt solution
IP	immunoaffinity precipitation
NTA	nanoparticle tracking analysis
PI	protease inhibitor
SEC	size exclusion chromatography
UC	ultracentrifugation
UF	ultrafiltration
