# Supplementary material for: Extracellular Vesicle Separation Techniques Impact Results from Human Blood Samples: Considerations for Diagnostic Applications
Source: Int J Mol Sci. 2021 Aug 26;22(17):9211. doi: 10.3390/ijms22179211 (PMC8431127; doi:10.3390/ijms22179211)
Supplement: Supplementary file 1 [file ijms-22-09211-s001.zip › ijms-1338443-supplementary.pdf]

Figure S1

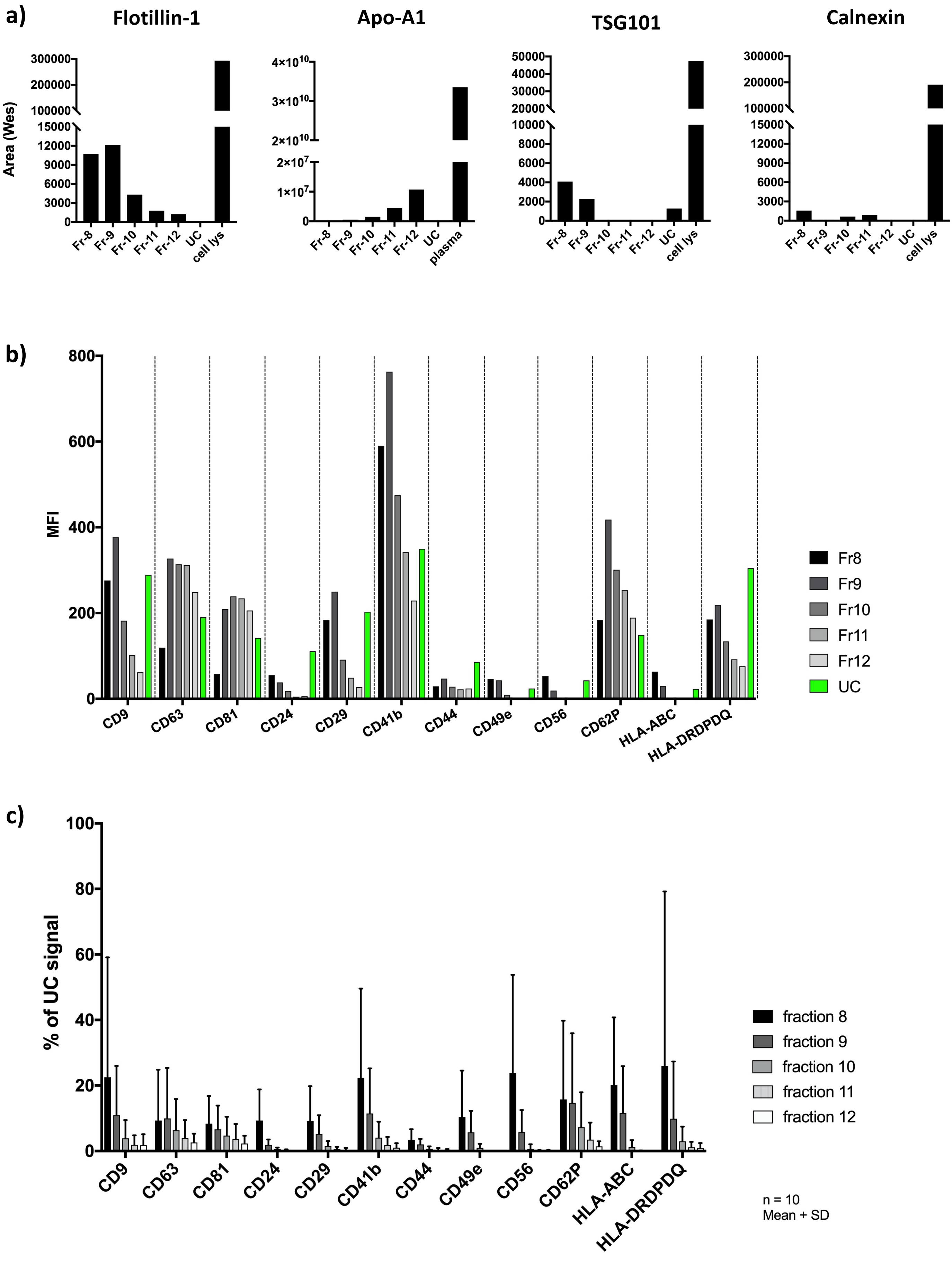
a)
Flotillin-1
Apo-A1
TSG101
Calnexin
Area (Wes)
Fr-8
Fr-9
Fr-10
Fr-11
Fr-12
UC
cell lys
plasma
b)
MFI
Fr8
Fr9
Fr10
Fr11
Fr12
UC
CD9
CD63
CD81
CD24
CD29
CD41b
CD44
CD49e
CD56
CD62P
HLA-ABC
HLA-DRDPDQ
c)
% of UC signal
fraction 8
fraction 9
fraction 10
fraction 11
fraction 12
n = 10
Mean + SD

Figure S1

SEC- and UC-EV: Quantified immunodetection and MFI values from MACSplex assay

(a) Immunodetection of flotillin-1, Apo-A1, TSG101, and calnexin illustrated as calculated area (analyzed with Compass software, ProteinSimple), reflecting the signal intensity. (b) Surface protein analysis with the MACSPlex assay. Depicted is the mean fluorescence intensity (MFI) of one representative experiment out of 10. (c) Surface protein analysis with the MACSPlex assay. Depicted is the mean of 10 experiments as a percentage of the normalized signal from the UC-EVs.

Fr-8 to 12: collected fraction from SEC column, SEC: size exclusion chromatography, UC: ultracentrifugation

Figure S2

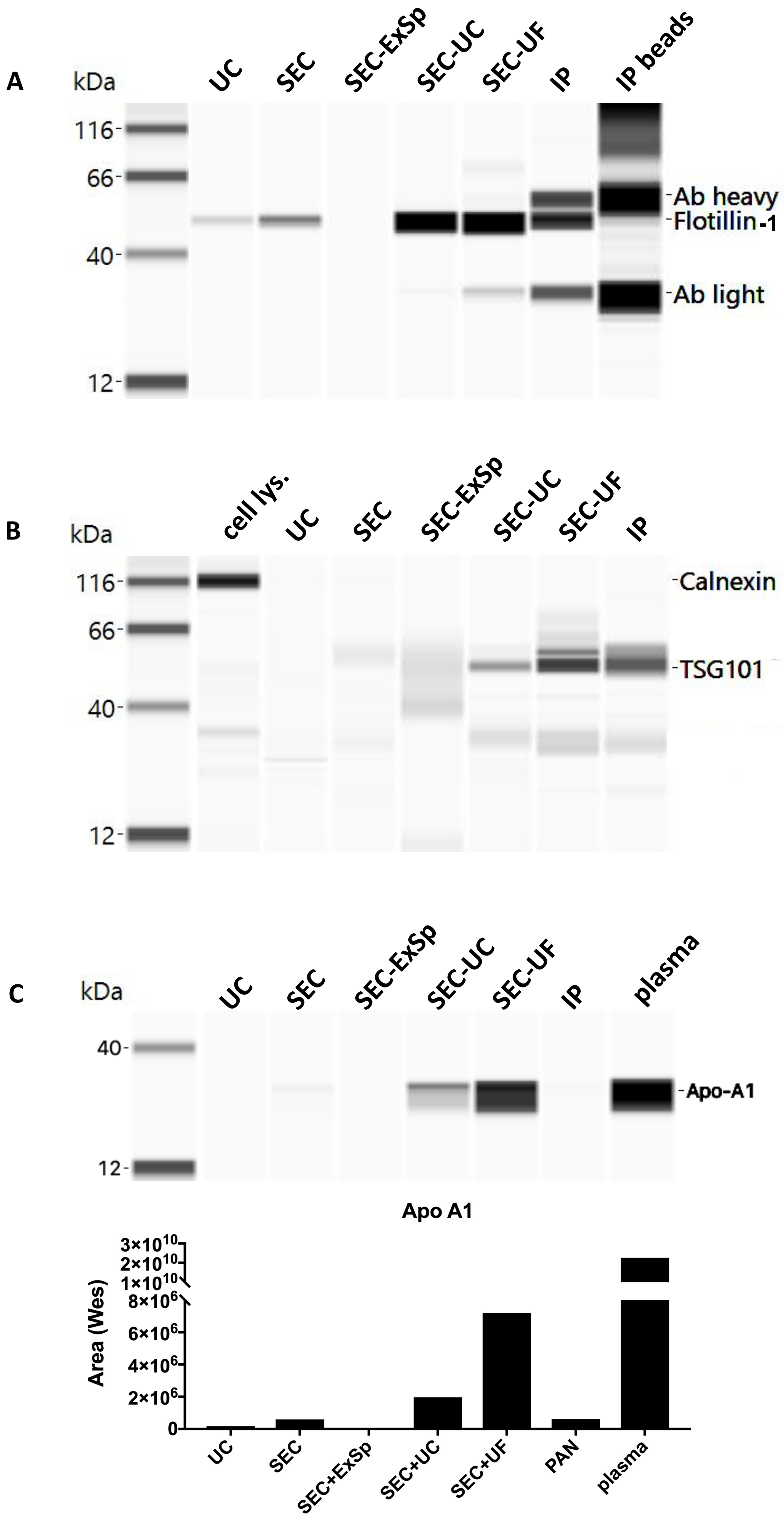
A
kDa
UC
SEC
SEC-ExSp
SEC-UC
SEC-UF
IP
IP beads
116
66
40
12
Ab heavy
Flotillin-1
Ab light
B
kDa
cell lys.
UC
SEC
SEC-ExSp
SEC-UC
SEC-UF
IP
116
66
40
12
Calnexin
TSG101
C
kDa
UC
SEC
SEC-ExSp
SEC-UC
SEC-UF
IP
plasma
40
12
Apo-A1
Apo A1
Area (Wes)
3×10^10
2×10^10
1×10^10
8×10^6
6×10^6
4×10^6
2×10^6
0
UC
SEC
SEC+ExSp
SEC+UC
SEC+UF
PAN
plasma

Figure S2 - **EV markers can be detected in all samples but SEC-ExSp-EV**

Immunodetection of the EV marker (A) flotillin-1 and (B) TSG101 and the ER marker calnexin, and (C) apolipoprotein A1 (Apo-A1) shown by Simple Western. The graph illustrates the calculated area (analyzed with Compass for SW software, ProteinSimple), reflecting the signal intensity.

ExSp: precipitation using Exo-spin^{TM} exosome purification kit, IP: immunoaffinity precipitation, SEC: size exclusion chromatography, UC: ultracentrifugation, UF: ultrafiltration

Figure S3

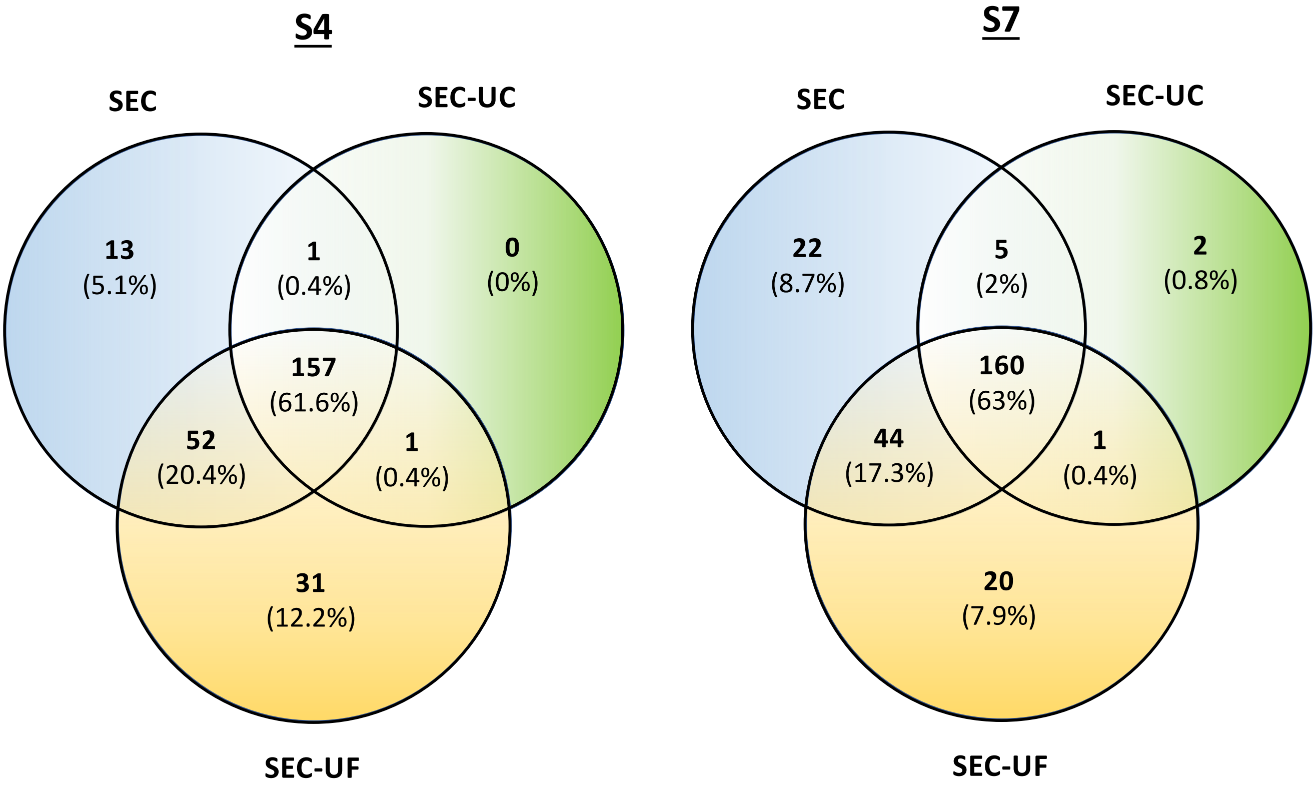

Figure S3

Venn diagram of proteins detected in EV derived from serum samples 4 and 7, which were isolated with SEC alone or followed by concentration with UC and UF. A high overlap of identified proteins (62.3%) was observed in samples irrespective of the isolation method. Concentration of EVs after SEC isolation results in the identification of up to 12% additional proteins.

Table S1 - Details on parameters of proteomic analysis

a)

**Reversed phase liquid chromatography (RPLC)**	
instrument	Ultimate 3000 RSLC (Thermo Fisher Scientific)
trap column	75 µm inner diameter, packed with 3 µm C18 particles (Acclaim PepMap100, Thermo Fisher Scientific)
analytical column	Accucore 150-C18, (Thermo Fisher Scientific)25 cm x 75 µm, 2,6 µm C18 particles, 150 Å pore size
buffer system	binary buffer system consisting of 0.1% acetic acid, (buffer A) and 100% ACN in 0.1% acetic acid (buffer B)
flow rate	300 nl/min
gradient	linear gradient of buffer B from 2% up to 25%
gradient duration	120 min
column oven temperature	40°C

b)

**Mass spectrometry (MS/MS)**	
instrument	Q Exactive plus mass spectrometer (Thermo Fisher Scientific)
electrospray	Nanospray Flex Ion Source
operation mode	data-independent
Full MS	
MS scan resolution	70,000
AGC target	5e6
maximum ion injection time for the MS scan	120 ms
Scan range	300 to 1650 m/z
Spectra data type	profile
dd-MS2	
Resolution	35,000
MS/MS AGC target	3e6
maximum ion injection time for the MS/MS scans	auto
Spectra data type	profile
selection for MS/MS	1
isolation window	see below
Fixed first mass	-
dissociation mode	higher energy collisional dissociation (HCD)
normalized collision energy	27.5

Table S1

c)

window	m/z	window size [m/z]
1	400-430	30
2	428-459	31
3	457-483	26
4	481-506	25
5	504-531	27
6	529-554	25
7	552-576	24
8	574-600	26
9	598-624	26
10	622-650	28
11	648-676	28
12	674-704	30
13	702-735	33
14	733-771	38
15	769-810	41
16	808-856	48
17	854-914	60
18	912-1000	88
19	998-1220	222

Table S1
Details on parameters of proteomic analysis for a) reversed phase liquid chromatography, b) mass spectrometry and c) data independent acquisition (DIA) with 19 different windows.

Table S2

miR	Assay ID	Mature miRNA sequence
hsa-miR-103a-3p	478253_mir	5‘-AGCAGCAUUGUACAGGGCUAUGA-3‘
hsa-miR-484	478308_mir	5‘-UCAGGCUCAGUCCCCUCCCGAU-3‘
hsa-miR-128-3p	477892_mir	5‘-UCACAGUGAACCGGUCUCUUU-3‘
hsa-miR-23a-3p	478532_mir	5‘-AUCACAUUGCCAGGGAUUUCC-3‘
hsa-miR-92b-3p	477823_mir	5‘-UAUUGCACUCGUCCCGGCCUCC-3‘
hsa-miR-106a-5p	478225_mir	5‘-AAAAGUGCUUACAGUGCAGGUAG-3‘
hsa-miR-199a-3p	477961_mir	5‘-ACAGUAGUCUGCACAUUGGUUA-3‘
hsa-let-7a-5p	478575_mir	5‘-UGAGGUAGUAGGUUGUAUAGUU-3‘

Table S2
miRNA assays and sequences used for qRT-PCR (Thermo Fisher Scientific; Waltham, MA, USA).
